# Physiological response of microalga *Dunaliella parva* when treated with MeJA, GA3

**DOI:** 10.1371/journal.pone.0308730

**Published:** 2024-10-22

**Authors:** Lingru Ruan, Lina Wu, Yanyan Liang, Bingbing Pang, Changhua Shang

**Affiliations:** Key Laboratory of Ecology of Rare and Endangered Species and Environmental Protection (Guangxi Normal University), Ministry of Education & Guangxi Key Laboratory of Landscape Resources Conservation and Sustainable Utilization in Lijiang River Basin, Guangxi Normal University, Guilin, Guangxi, China; Sathyabama Institute of Science and Technology, INDIA

## Abstract

DpAP2 is a transcription factor regulating carotenoid biosynthesis pathway. It was speculated that MeJA significantly decreased expression of *DpAP2* gene, then the decreasing *DpAP2* expression significantly inhibited expression of some key enzyme genes such as *PSY*, *PDS* and *GGPS* in carotenoid biosynthesis pathway. In contrast, it was speculated that GA3 significantly increased expression of *DpAP2* gene, then the increasing *DpAP2* expression significantly increased expression of some key enzyme genes such as *PDS* and *GGPS* in carotenoid biosynthesis pathway. To increase the content of carotenoid, we evaluated the effect of *DpAP2* overexpression on carotenoid accumulation in *D*. *parva*. Transgenic *D*. *parva* showed a higher carotenoid content (3.18 mg/g DW) compared with control group (2.13 mg/g DW) at 9 d. The dosage effects of exogenous hormones MeJA and GA3 were found in *D*. *parva* cells treated with different concentrations of MeJA (10, 20, 50, 100 μM) and GA3 (10, 20, 50, 100 μM). The high concentrations of MeJA (10–100 μM) inhibited the accumulation of carotenoid, and the relative expression of *DpAP2*, *PSY*, *PDS* and *GGPS* decreased significantly. On the contrary, the relative expression of *DpAP2*, *PDS* and *GGPS* increased significantly when *D*. *parva* was treated with 10, 20, 50 and 100 μM GA3, which promoted the biosynthesis of carotenoid. Therefore, we inferred that there was a hierarchical regulation from hormone, transcription factor, key enzyme gene to carotenoid accumulation in carotenoid biosynthesis. Carotenoid biosynthesis was enhanced by *DpAP2* overexpression (1.4930 fold of control) and exogenous substances such as GA3 (1.5889 fold of control), which laid a foundation for massive accumulation of carotenoids in microalgae. In the future, further studies were required to demonstrate the complex regulatory network.

## 1. Introduction

Carotenoids are a kind of important bioactive substances. Carotenoids are mainly used as colorants, feed supplements, nutraceuticals, health care products and cosmetics **[1]**. The process of carotenoid biosynthesis is a greatly complex pathway. Current researches have paid high attention to the regulatory factors of metabolic pathway such as transcription factors (TFs) **[2]**. A great number of TFs involved in carotenoid biosynthesis pathway were reported in higher plants, which included NAC, MADS-box and AP2/ERF **[3–5]**. Various studies have shown that AP2 is involved in the development, carotenoid metabolism, growth, and responses to various stresses **[6–8]**. MdAP2-34 improved carotenoid accumulation by activating *MdPSY2-1* promoter in apple **[5]**. Sang et al. found that AP2a mediated ethylene signaling to regulate brassinosteroids synthesis and signaling in tomato fruits **[9]**. Brassinosteroids played important roles in lycopene (a kind of carotenoids) synthesis after beginning of fruit ripening. Our unpublished study found that DpAP2 regulated target genes related to carotenoid biosynthesis by ChIP-Seq analysis in *D*. *parva*. However, at present the effect of *DpAP2* overexpression on carotenoid accumulation is unclear in *D*. *parva*.

Our previous study has elucidated the biosynthetic pathway of carotenoids **[10]**. In brief, carotenoids are synthesized by several steps including desaturation, cyclization, hydroxylation and epoxidation within plastids. Some important enzymes include GGPS, PSY, PDS in carotenoid biosynthesis pathway. Although overproduction of carotenoids has been extensively studied in microalgae, its regulatory mechanism remains unclear, which needs to be further elucidated **[11]**. A large number of studies have elucidated the role of structural genes encoding enzymes of carotenoid biosynthetic pathway in higher plant, but it remains unclear regarding what genes and enzymes primarily involve in the regulation of carotenoids accumulation in microalgae **[10, 12]**. Our laboratory conducted a series of studies about genes in carotenoid biosynthesis such as *GGPS*, *PSY*, *PDS*. Analysis of *DpGGPS* expression showed a correlation between *DpGGPS* expression and the shift of NaCl concentration **[13]**. In addition, the inconsistency of changing trend between *DpGGPS* expression and carotenoids content showed the complexity of the regulatory network of carotenoid biosynthesis in *D*. *parva*. Furthermore, the effects of NaCl concentration up-shock and nitrogen limitation on carotenoids content and expression of *PSY* and *PDS* were explored in *D*. *parva* [a7]. The results suggested that both NaCl concentration up-shock and nitrogen limitation significantly affected carotenoids content and expression of *PSY* and *PDS* in *D*. *parva* **[14]**. The above studies revealed the importance of three genes including *GGPS*, *PSY* and *PDS* in carotenoids accumulation of *D*. *parva*.

GA3 plays an important role in plant growth and development, stress responses and inhibition of chlorophyll degradation **[15]**. Studies showed that GAMYB was an MYB-type transcription factor regulated by GA3, which was involved in plant growth and development, and biomass synthesis **[16]**. Sagawa et al. identified an R2R3-MYB transcription factor, Reduced Carotenoid Pigmentation 1, that positively regulated carotenoid biosynthesis during flower development of *Mimulus lewisii* **[17]**. Genome-wide comparative analysis of the R2R3-MYB gene family in five solanaceae species identified members (*Lba11g0183* and *Lba02g01219*) regulating carotenoid biosynthesis in wolfberry **[18]**. However, the effect of GA3 on *D*. *parva* is unclear at present.

MeJA is a natural plant growth regulator that plays a crucial role in plant signaling networks, regulating plant damage response, growth and development, and many other physiological processes **[19]**. MeJA can promote the synthesis of carotenoids in various plant. However, MeJA can inhibit the synthesis of carotenoids in some cases. Exogenous substance MeJA had a dose effect. A low concentration of MeJA promoted the synthesis of carotenoids, while a high concentration of MeJA inhibited the accumulation of carotenoids **[20]**. However, the effect of MeJA on *D*. *parva* is unclear at present.

To elucidate the mechanism of carotenoid biosynthesis and increase the content of carotenoids, we evaluated the effect of *DpAP2* overexpression on carotenoids accumulation in *D*. *parva* cells. Moreover, physiological response was investigated under the treatment of hormones like GA3 and MeJA in *D*. *parva*. This study laid a foundation for enhancing the expression of multiple genes in carotenoid biosynthesis pathway by manipulating the expression of transcription factor gene (*DpAP2*) and for understanding the effects of exogenous substances (GA3 and MeJA) on carotenoid synthesis in the future.

## 2. Materials and methods

### 2.1. Microalga and growth condition

*D*. *parva* FACHB-815 was purchased from Freshwater Algae Culture Collection at the Institute of Hydrobiology (Wuhan, China). *D*. *parva* were cultured in Dm medium based on our previous study **[10, 14]**.

### 2.2. Cloning of *DpAP2* promoter

Three gene-specific primers NEW-SP1, NEW-SP2 and NEW-SP3 (**S1 Table**) were designed according to *DpAP2* sequence (GenBank Accession no. ON548537). The genomic DNA of *D*. *parva* was extracted by CTAB method, then used for 1st PCR of genome walking **[21]**. PCR (1st PCR, 2nd PCR, 3rd PCR) was performed according to amplification conditions of Genome Walking Kit (Takara, Dalian, China). The obtained promoter sequence was analyzed by PlantCARE to predict its *cis*-acting elements.

### 2.3. Construction of recombinant expression vector pBI221-UbiΩ-CAT-DpAP2

To obtain the complete *DpAP2* sequence, a pair of primers AP2-CHN-BamHI and AP2-CHC-BsrGI containing *Bam*H I and *Bsr*G I sites were designed (**S1 Table**). Then *DpAP2* sequence was amplified by PCR and cloned into T-Vector pMD19 (Simple). The target gene *DpAP2* was further cloned into the expression vector pBI221-GFP-UbiΩ-CAT from the recombinant T-Vector pMD19 (Simple) by restriction enzymes (*Bam*H I and *Bsr*G I). The 30 μL enzyme reaction mixture included 0.75 μL *Bam*H I, 0.75 μL *Bsr*G I, 3 μL 10XT Buffer, 3 μL 0.1% BSA, 5 μL the recombinant T-Vector pMD19 (Simple), and 17.5 μL ddH_2_O, which was incubated at 37°C for 30 min. After enzymatic digestion, the final *DpAP2* product (2331 bp) was purified and cloned into the expression vector pBI221-GFP-UbiΩ-CAT using DNA Ligase <LONG> kit (Takara, Dalian). Finally, the recombinant expression vector pBI221-UbiΩ-CAT-DpAP2 was obtained.

### 2.4. Construction of *DpAP2*-overexpressing transgenic *D*. *parva*

The transformation method of *D*. *parva* was based on our published study **[22, 23]**. *D*. *parva* was cultured in Dm medium until the logarithmic growth stage (nearly one or two weeks). Then 10 mL algal cells were centrifuged at 4000 rpm for 5 min at 4°C, and washed with 10 mL TE buffer. 500 μL TE buffer and the same volume of LiA_C_ (0.2 M) were added for resuspending precipitation. The extracted plasmids (pBI221-UbiΩ-CAT-DpAP2, 100 μL, 600 ng/μL) was added to the above mixture. Then the mixture was kept at 29°C for 30 min. 1 mL of 70% PEG-4000 was added, and kept at 29°C for 1 h.10 mL of autoclaved seawater was added to the mixture and centrifuged at 4000 rpm for 10 min. The supernatant was discarded, and the precipitate was resuspended with 10 mL of Dm medium with chloramphenicol (final concentration of 800 ng/μL), and cultured at 25°C. To verify the transgenic *D*. *parva*, plasmids were isolated from transgenic *D*. *parva* and control, and used for PCR amplification as templates by primers pBI221-N and pBI221-C (two universal primers for the vector pBI221-GFP-UbiΩ-CAT, **S1 Table**). The transgenic *D*. *parva* was inoculated into Dm medium with chloramphenicol (60 ng/μL).

### 2.5. Experimental design

The dose of microalgal mother inoculum was 10–20% (v/v). The mother cultures were inoculated into Dm medium with different concentrations of MeJA (10, 20, 50, 100 μM) and GA3 (10, 20, 50, 100 μM), respectively. The cells were cultured for 13 d. Cell density, pigment content, carbohydrate content, protein content and mRNA level were determined.

### 2.6. Determination of physiological and biochemical indicators

Determination of cell density, contents of carotenoid, chlorophyll were performed according to our previous study **[22]**. Total carbohydrate content was determined by anthrone colorimetry **[24]**.

### 2.7. Fluorescence quantitative PCR (FQ-PCR) analysis

To determine the expression of several key genes, including *DpAP2*, *PSY*, *PDS* and *GGPS*, FQ-PCR was performed using the corresponding primers (**S1 Table**). The relative expression of key genes was determined using the copies ratio of these genes and *D*. *parva Actin* gene.

### 2.8. Statistical analysis

The measured data were expressed as mean±standard error (SE) according to three independent experiments. Statistical analysis was performed using software SPSS 10.0. Analysis of variance (ANOVA) with Duncan’s multiple range test was used to compare significance level **[11]**.

## 3. Results and discussion

### 3.1. Construction of transgenic *D*. *parva* and the effect of *DpAP2* overexpression on carotenoid content

The positive *E*. *coli* DH5α colonies were verified via PCR by the specific primers AP2-CHN-BamH I and AP2-CHC-BsrGI (**S1 Table**). Electrophoresis showed a 2349 bp band which was identical to *DpAP2* sequence (GenBank Accession no. ON548537) (**S1 Fig**). **S1 Fig** indicated that overexpression vector pBI221-UbiΩ-CAT-DpAP2 was successfully constructed. The recombinant overexpression vector was transformed to *D*. *parva*, and the algal transformants were screened. The results showed that control group died under chloramphenicol stress, however, the growth of transgenic *D*. *parva* was normal (**S2 and S3 Figs**). Plasmids were isolated from transgenic *D*. *parva* and control as templates of PCR amplification. The success of transgenic *D*. *parva* containing pBI221-UbiΩ-CAT-DpAP2 was verified by PCR using primers pBI221-N and pBI221-C. As shown in S4 Fig, the presence of predicted bands (about 2674 bp) showed the success of transgenic *D*. *parva* with the overexpression of *DpAP2* gene (**S4 Fig**).

**Fig 1:**
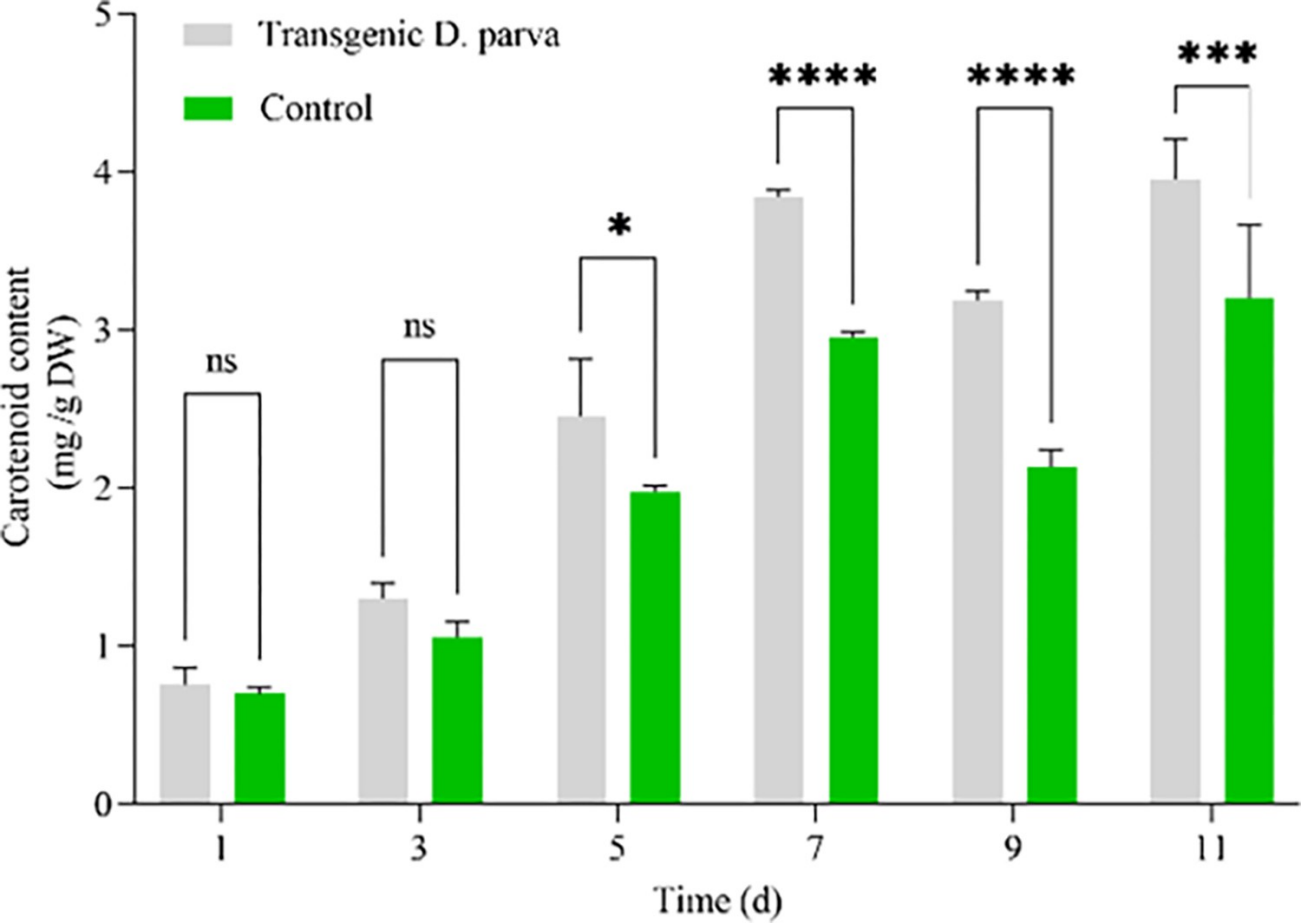
Carotenoid contents of control and transgenic *D*. *parva*.

Carotenoid contents of control and transgenic group were shown in **Fig 1**. Carotenoid contents increased from 1 d to 7 d, but slightly declined at 9 d for control and transgenic group. Carotenoid content of transgenic group reached 3.18 mg/g DW which increased by 49.30% compared with carotenoid content of control (2.13 mg/g DW) at 9 d. **Fig 1** suggested that *DpAP2* could promote carotenoid synthesis in transgenic *D*. *parva*.

TFs played vital regulatory roles during growth, development and response to a variety of environmental stresses **[25]**. The complex TF family AP2/ERF can efficiently regulate carotenoid biosynthesis in plants. The TFs (AP2, AP2-like) potentially affect carotenoid biosynthesis in *Prunus armeniaca* fruit **[26]**. In apple, MdAP2-34 could improve carotenoid content via binding to *MdPSY2-1*

promoter **[5]**. However, there were few reports about the effect of microalgal *AP2* gene on carotenoid content in microalgae. We successfully cloned *DpAP2* gene, constructed its transgenic *D*. *parva*, and firstly reported the increase in carotenoid content due to *DpAP2* overexpression in *D*. *parva*.

### 3.2. Cloning and analysis of *DpAP2* promoter

The *DpAP2* promoter (1921 bp) was obtained by Genome Walking method (**S5 Fig**). Its sequence (GenBank Accession no. PP797581) was shown in **S6 Fig**. The potential *cis*-acting elements in *DpAP2* promoter were predicted by PlantCARE online software. Several light-responsive elements including G-Box, L-box and TCCC-motif were predicted to regulate the transcriptional activity of *DpAP2* after light-induction. In addition, this promoter also had some predicted response elements of hormones such as abscisic acid, gibberellin, methyl jasmonate, and drought stress (**Table 1**). The prediction indicated that various environmental factors might regulate *DpAP2* expression at the transcriptional level.

**Table 1:** Predicted *cis*-acting elements in *DpAP2* promoter.

Name	Position	Function
ABRE	+677, +1437, +295, +674, -676, -498	involved in the abscisic acid response
CAAT-box	+29, +1230, -723, +1774, -416, +1585, -1037, -1838, +137, -1300, -623, -1713	common element in promoter and enhancer regions
CCAAT-box	+88	MYBHv1 binding site
CGTCA-motif	+1603, -102	involved in MeJA response
G-Box	+676, -1436	involved in light response
G-box	-445, +676, +498, +1434	involved in light response
P-Box	-1169	gibberellin response
TATA-box	-1515	A core element near transcription start point
L-Box	+699	involved in light response
TCCC-motif	+1745	involved in light response
TGACG-motif	+102, -1603	involved in MeJA response
MBS	-559, -1758	MYB binding site, involved in drought-inducibility

### 3.3. Growth of *D*. *parva*

**Fig 2:**
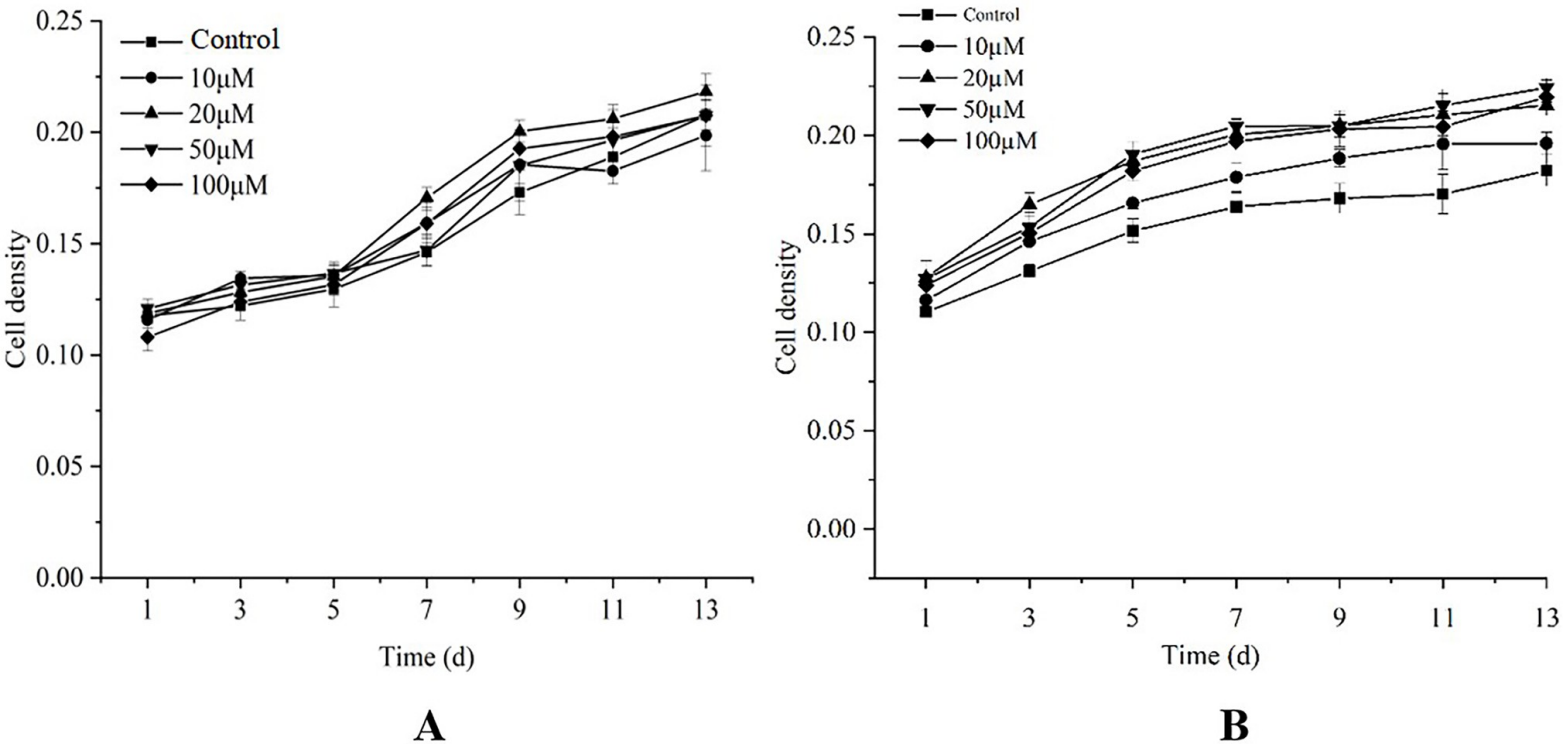
Cell density of *D*. *parva* under different concentrations of MeJA (A) and GA3 (B).

Cell density of *D*. *parva* treated with different concentrations of exogenous substances (MeJA and GA3) was determined. The highest cell density (0.2003) of 10 μM MeJA treated group was significantly higher than that of control group (0.1680) at 9d, which increased by 19.22% (Fig 2A). The highest cell density (0.2152) of 50 μM GA3 treated group was significantly higher than that of control group (0.1702) at 11 d (Fig 2B).

The addition of MeJA in *Taxus* cultures improved paclitaxel accumulation, but suppressed growth by inhibiting cell cycle progression **[27]**. Treating seed using MeJA could enhance resistance to rice water weevil but decrease plant growth in rice **[28]**. GA3 completely compensated the obvious inhibition of root growth caused by a gibberellin biosynthesis inhibitor (uniconazole P) **[29]**. GA3- enriched biochar is an effective treatment for alleviating drought and Cd stress in wheat, which resulted in a significant increase in shoot length (44.99%) and shoot fresh weight (63.59%) over control **[30]**. The above studies indicated that GA3 could improve plant growth, but MeJA decreased plant growth. In our present study, both MeJA and GA3 improved the growth of *D*. *parva*, perhaps the effect of hormones had species specificity.

### 3.4. Carotenoid content

**Fig 3:**
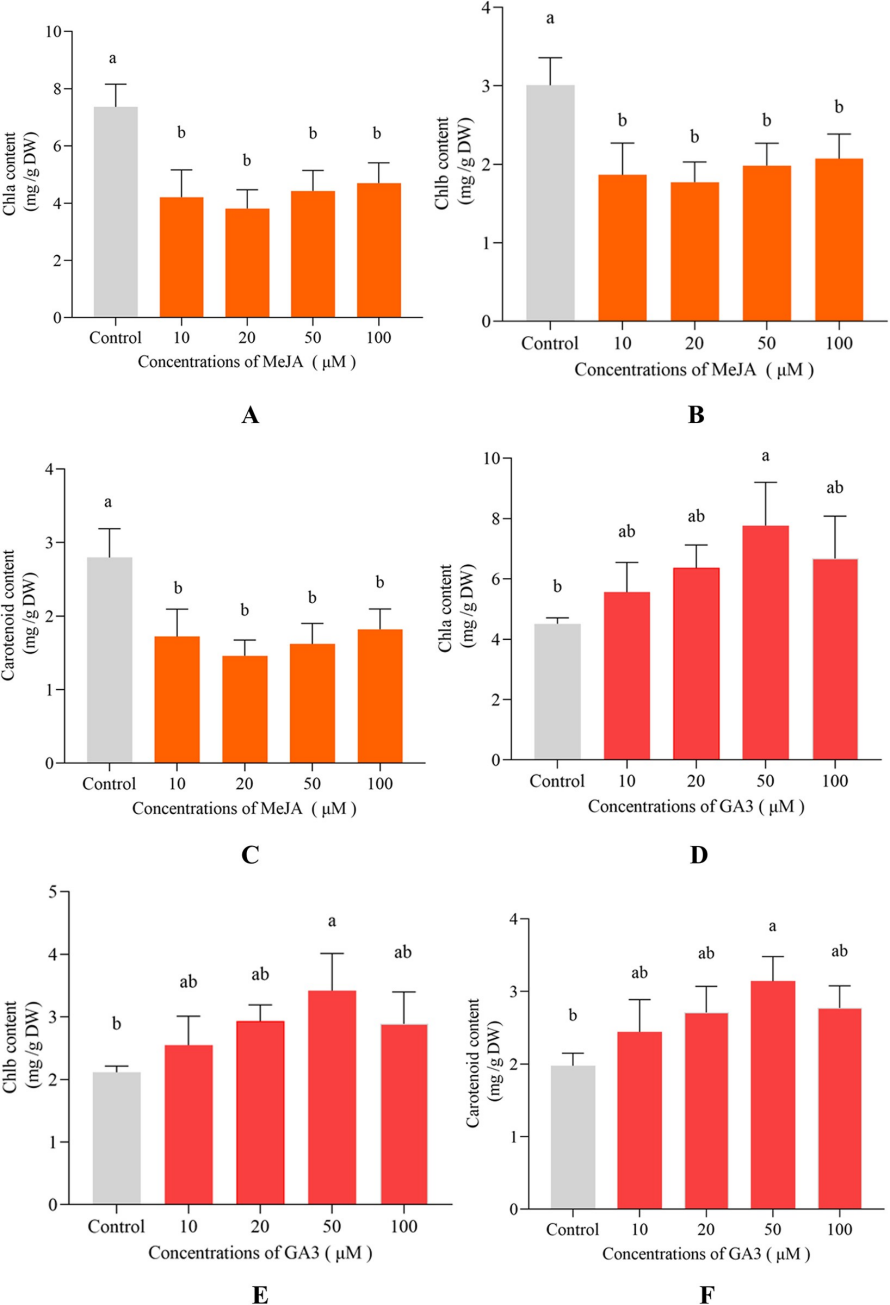
Contents of chlorophyll a, chlorophyll b (Chl a, Chl b) and carotenoid in *D*. *parva* cells under treatment of various concentrations of MeJA and GA3.

Synthesis of Chlorophyll a (Chl a), Chlorophyll b (Chl b) and carotenoid will influence some metabolic processes such as photosynthesis, their contents were evaluated at 13 d (**Fig 3**) **[31]**. Carotenoid contents of algal cells decreased significantly under MeJA treatment. Carotenoid contents were 1.72 mg/g DW, 1.46 mg/g DW, 1.62 mg/g DW and 1.82 mg/g DW under the treatment of different MeJA concentrations (10 μM, 20 μM, 50 μM, 100 μM), which decreased by 38.39%, 47.80%, 41.99% and 34.87% compared with carotenoid content of control group at 13 d (**Fig 3C**). In addition, the contents of Chla and Chlb in 100 μM MeJA treated group decreased by 36.15% and 31.12% compared with control (**Fig 3A and 3B**). On the contrary, carotenoid content reached 3.14 mg/g DW under 50 μM GA3 treatment at 13 d, which was 58.89% higher than that of control group (**Fig 3F**). Meanwhile, the contents of Chl a (7.77 mg/g DW) and Chl b (3.42 mg/g DW) of 50 μM GA3 treated groups were much more than that of control group (4.51 mg/g DW, 2.11 mg/g DW), which increased by 72.27% and 61.67%, respectively (**Fig 3D and 3E**).

After MeJA treatment, rice greatly responded to oxidative stress, and decreased chlorophyll content and photosynthetic efficiency **[32]**. Both MeJA- and norflurazon-treated *Arabidopsis* showed destruction of Chls and a substantial decrease in photosynthetic activity **[33]**. Exogenous addition of MeJA decreased chlorophyll content, carbon assimilation rate and destroyed membrane integrity, and a key factor (tomato MYC2) was related to MeJA-induced leaf senescence **[34]**. MeJA is plant growth regulator for improving carotenoid accumulation in yellow maize sprouts via upregulation of expression of key genes involved in carotenoid biosynthesis pathway **[35]**. The results of Divya et al. indicated that MeJA induced-signaling network stimulated expression of carotenogenic genes, leading to carotenoid accumulation in coriander (*Coriandrum sativum* L.) foliage **[36]**. The above results differed from our present study. In our study, Chls content didn′t change, and carotenoid content decreased. Perhaps the effects of MeJA on the contents of Chls and carotenoid had species specificity.

GA3 enriched deashed biochar alleviated cadmium and drought stresses in wheat via the improvement of growth and chlorophyll contents **[30]**. Chlorophyll content per surface unit of the leaf decreased, and the decrease was proportional to the increase in GA3 concentration due to GA3 treatment (0.01, 1, 10 and 100 ppm) in *Phaseolus vulgaris* L. **[37]**. With treatments of 20 mg/L and 40 mg/L GA3, the expression of carotenoid genes (such as *ipi-1*, *ipi-2*, *psy*, *pds* and *bkt2*) was enhanced, leading to higher astaxanthin accumulation in unicellular alga *Haematococcus pluvialis* **[38]**. Total carotenoid content obviously decreased with the increase in concentrations of GA3, ABA and NaCl in the callus of *Scutellaria baicalensis* Georgi because these substances affected the expression of genes related to carotenoid biosynthetic pathway **[39]**. To sum up, the effects of GA3 on the contents of Chls and carotenoid had obvious species specificity. In our present study, GA3 treatment substantially improved the contents of Chls and carotenoid in *D*. *parva*.

### 3.5. Carbohydrate content

**Fig 4:**
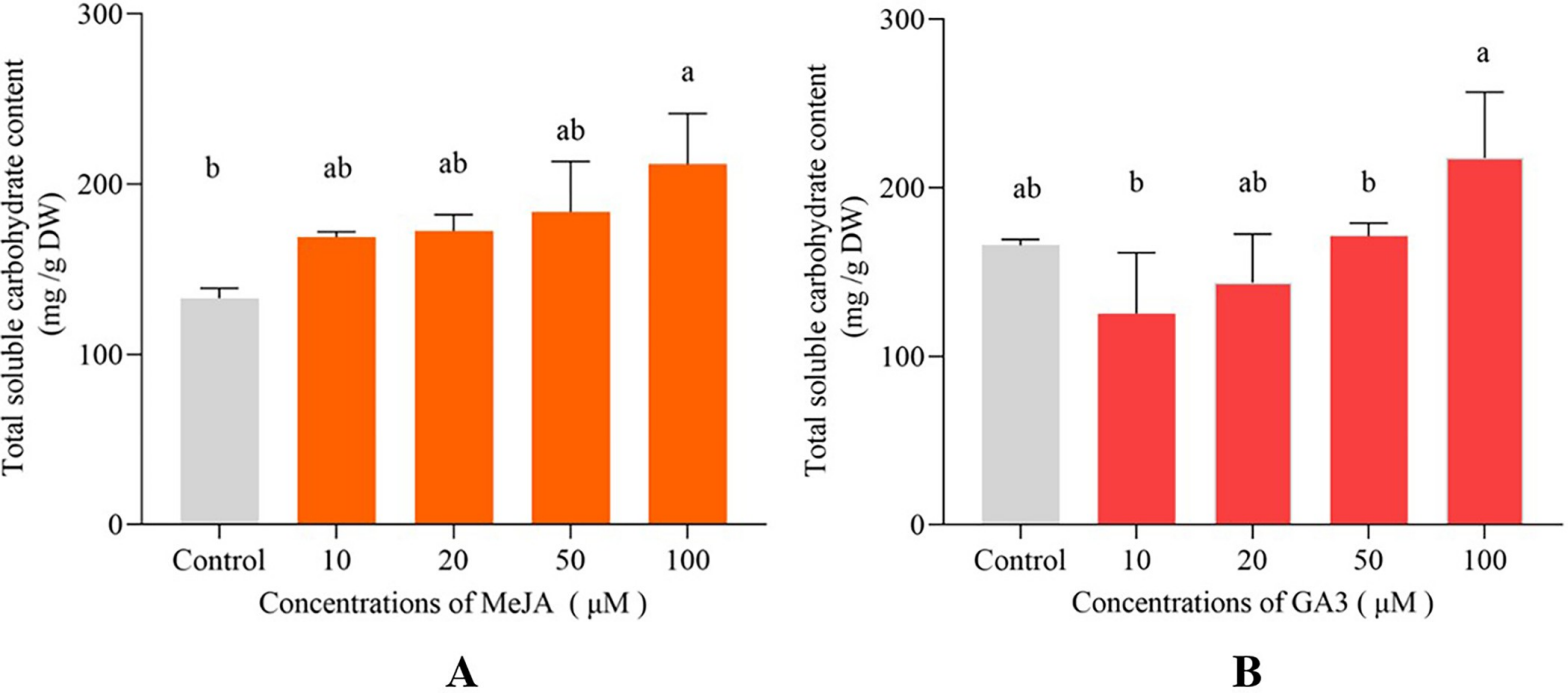
Total carbohydrate contents of *D*. *parva* cells with the treatment of MeJA (A) and GA3 (B).

Carbohydrate contents were significantly enhanced with the treatment of 100 μM MeJA and 100 μM GA3. Carbohydrate content of 100 μM MeJA treated group could reach 211.50 mg/g DW which increased by 59.20% compared with carbohydrate content of control group (**Fig 4A**). Meanwhile, carbohydrate content of 100 μM GA3 treated group could reach the highest value (217.58 mg/g DW) which increased by 31.20% compared with carbohydrate content of control group (**Fig 4B**). However, there was no the obvious increase in carbohydrate content among other GA3 treated groups and control group (**Fig 4B**).

### 3.6. FQ-PCR analysis

**Fig 5:**
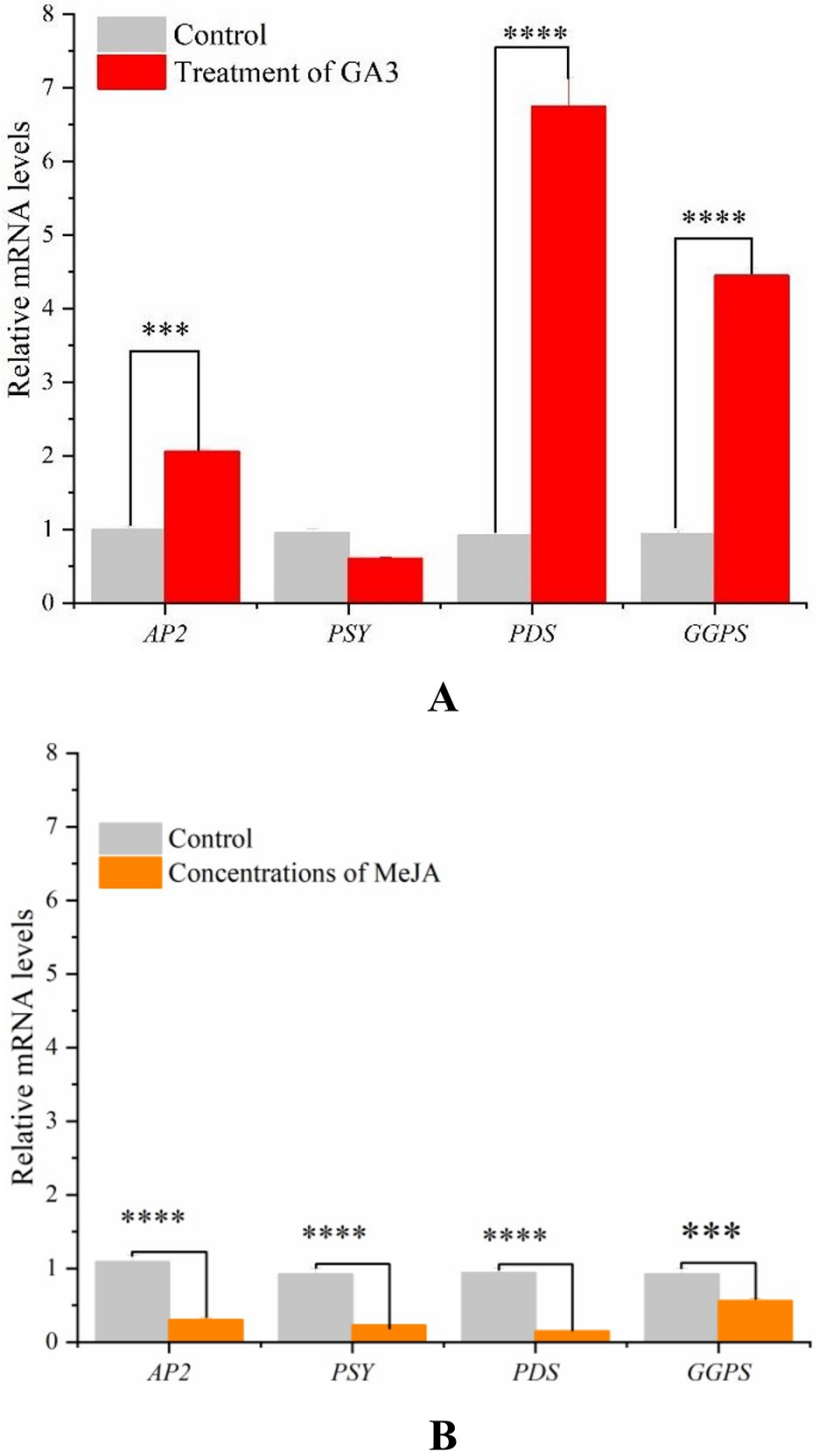
Relative mRNA levels of carotenogenic genes under the treatment of GA3 (A) and MeJA (B).

Previous studies had found that four key genes *AP2*, *PSY*, *PDS* and *GGPS* involved in the regulation of carotenoid synthesis pathway in plants, however, little was known about the function of four genes in *D*. *parva* **[5, 40, 41]**. In addition, AP2 is a transcription factor regulating carotenoid biosynthesis. The *GGPS*, *PSY* and *PDS* genes encode geranylgeranyl diphosphate synthase, phytoene synthase and phytoene desaturase, respectively. The mRNA levels of *DpAP2*, *PDS*, *GGPS* and *PSY* significantly decreased due to 10 μM MeJA treatment (**Fig 5B**). On the contrary, the relative expression of *DpAP2*, *PDS* and *GGPS* markedly increased when *D*. *parva* cells were treated with 50 μM GA3, which affected carotenoid biosynthesis (**Fig 5A**). Divya et al. found that MeJA induced carotenoid accumulation in coriander foliage by stimulating expression of carotenogenic genes **[36]**. Total carotenoid content obviously decreased with the increase in concentrations of GA3, ABA and NaCl in the callus of *Scutellaria baicalensis* Georgi due to the change in the expression of genes related to carotenoid biosynthetic pathway **[39]**. Combining previous perspectives and our present results, it was inferred that MeJA and GA3 firstly affected the expression of *DpAP2*, then DpAP2 regulated the transcription of key genes *PSY*, *PDS* and *GGPS* related to carotenoid biosynthetic pathway, leading to the change in carotenoid content.

The APETALA2/ethylene-responsive factor (AP2/ERF), is a superfamily, which contains four subfamilies (ERF, AP2, CBF/DREB and RAV) in plants **[42]**. Many researchers clarified that TFs involved in biomass production, response to various stresses such as salt and drought, and sesquiterpenoid biosynthesis **[20]**. Our present study showed that *DpAP2* had a close relationship with carotenoid biosynthesis. AP2 played a crucial regulatory role in carotenoid synthesis in tomato by directly activating the transcription of *DWF* gene with putative AP2-binding motifs (TTTGTT or AACAAA) **[9]**. In this study, *DpAP2* gene was overexpressed in *D*. *parva* cells, which showed higher carotenoid value content (3.18 mg/g DW) compared with control (2.13 mg/g DW) at 9 d, which indicated the essential role of DpAP2 in the regulation of carotenoid biosynthesis of *D*. *parva*. The binding site of DpAP2 will be detected by electrophoretic mobility shift assay in the future.

Hormones GA3 and MeJA play an important role in carotenoid metabolism in many plants. It was found that transcript levels of *PSY*, *PDS*, *ZDS*, *LCYB*, *LCYE*, *BCH1* and *CYP97C* significantly increased after treatment with 0.5 μM MeJA, and exogenous substance MeJA affected the synthesis of carotenoid **[43]**. However, excessive MeJA inhibited the accumulation of carotenoid. In this paper, different concentrations of MeJA (10, 20, 50, 100 μM) were set to treat algal cells. We found that MeJA had a dose effect, high concentrations of MeJA (10–100 μM) inhibited carotenoid accumulation. The relative expression of *PSY*, *PDS*, *GGPS* and *DpAP2* decreased significantly when *D*. *parva* was treated with high concentrations of MeJA, which inhibited carotenoid accumulation. GA3 plays an important role in plant growth and development, and inhibition of chlorophyll degradation. The former study had shown that GAMYB was a MyB-type transcription factor regulated by GA3, which was involved in plant growth and development, and biomass synthesis **[16]**. GID1 receptor protein (GA3 receptor) can sense GA3 signal and transmit it to plant growth inhibitor (DELLA protein), resulting in their degradation through ubiquitin-proteasome pathway. The interaction of proteins DELLA and GAMYB affects the expression of genes associated with carotenoid synthesis pathway (*LCYB*, *CRTISO*, *PDS*, *ZDS* and *BCH*), thereby promotes carotenoid accumulation. The relative mRNA levels of *PDS*, *DpAP2* and *GGPS* increased significantly under GA3 treatment, which promoted the accumulation of carotenoid in our present study. it was inferred that MeJA and GA3 firstly affected the expression of *DpAP2*, then DpAP2 regulated the transcription of key genes *PSY*, *PDS* and *GGPS* related to carotenoid biosynthetic pathway, leading to the change in carotenoid content.

## 4. Conclusion

Carotenoids are natural antioxidants, which play a key role in the photosynthetic metabolic pathway in the case of light damage caused by excessive light. The results showed that carotenoid biosynthesis was enhanced by transforming endogenous gene *DpAP2* into *D*. *parva*. Carotenoid content of transgenic *D*. *parva* reached 3.18 mg/g DW, which was 49.30% higher than that of control group (2.13 mg/g DW). MeJA had a dose effect, high concentrations of MeJA (10–100 μM) inhibited carotenoid accumulation. The relative mRNA levels of *PSY*, *PDS*, *GGPS* and *DpAP2* decreased significantly when *D*. *parva* was treated with high concentrations of MeJA. On the contrary, the relative expression of *DpAP2*, *PDS* and *GGPS* increased significantly under the treatment of GA3 (10–100 μM), which promoted carotenoid synthesis. Carotenoid biosynthesis was enhanced by *DpAP2* overexpression (1.4930 fold of control) and exogenous substance GA3 (1.5889 fold of control), which laid a foundation for improving the economic value of *D*. *parva* in the pharmaceutical industry.

## Supporting information

S1 Table The sequence of the primers. (DOCX)

S1 Fig Amplification of *DpAP2* full-length cDNA. Lane 1 and lane 2 indicate *DpAP2* full-length cDNA. Marker indicates DL2000 DNA marker.
(TIF)

S2 Fig Construction of transgenic *D*. *parva* overexpressing *DpAP2* gene. 1: Control group; 2: Control group containing chloramphenicol; 3/4: transgenic *D*. *parva* containing chloramphenicol.
(TIF)

S3 Fig Growth of transgenic *D*. *parva* and control after one month. The left culture was transgenic *D*. *parva*, and the right culture was control.
(TIF)

S4 Fig Verification of transgenic *D*. *parva*. Plasmids were isolated from transgenic *D*. *parva* and control as templates of PCR amplification. Lane 1 indicates transgenic *D*. *parva* group. Lane 2 indicates control group. Marker indicates DL2000 DNA marker.
(TIF)

S5 Fig Cloning of *DpAP2* promoter. Primers AP1/AP3 are degenerate primers from Genome Walking Kit. Marker indicates DL2000 DNA marker. AP1/AP3 indicate result for second PCR.
(TIF)

S6 Fig Sequence of *DpAP2* promoter. (TIF)

## A list of abbreviations

MeJA: methyl jasmonate
GA3: gibberellin A3
TF: transcription factor
NAC: NAM, ATAF1/2, CUC2
MADS-box: MCM1, AGAMOUS, DEFICIENS, SRF
AP2/ERF: APETALA2/ethylene-responsive factor
WT: wild type
DpAP2: *Dunaliella parva* AP2
D. parva: *Dunaliella parva*
ChIP-Seq: chromatin immunoprecipitation sequencing
DpGGP: *Dunaliella parva* GGPS
GGPS: geranylgeranyl diphosphate synthase
PSY: phytoene synthase
PDS: phytoene desaturase
MYB: : v-myb avian myeloblastosis viral oncogene homolog
GAMYB: gibberellin-regulated MYB
BSA: bovine serum albumin
1st PCR: first PCR
2nd PCR: second PCR
3rd PCR: third PCR
TE: Tris-EDTA
M: mol/L
SE: standard error
ANOVA: analysis of variance
MdAP2-34: *Malus domestica* AP2-34
MdPSY2-1: *Malus domestica* PSY2-1
Chl a: chlorophyll a
Chl b: chlorophyll b
DW: dry weight
ipi: isopentenyl diphosphate isomerase
bkt: β-carotene ketolase
ABA: abscisic acid
FQ-PCR: fluorescent quantitative PCR
ZDS: ζ-carotene desaturase
LCYB: lycopene β-cyclase
LCYE: lycopene epsilon-cyclase
BCH: non-heme carotene hydroxylase
CYP97C: carotenoid hydroxylase
CRTISO: carotenoid isomerase
GID1: GIBBERELLIN-INSENSITIVE DWARF1
DELLA: aspartic acid–glutamic acid–leucine–leucine–alanine
