# Supplementary material for: Physiological response of microalga Dunaliella parva when treated with MeJA, GA3
Source: PLoS One. 2024 Oct 22;19(10):e0308730. doi: 10.1371/journal.pone.0308730 (PMC11495637; doi:10.1371/journal.pone.0308730)
Supplement: S1 Table — (DOCX) [file pone.0308730.s001.docx]

**Supplementary Table S1 The sequence of the primers.**

| **Primer** | **Sequence (5′-3′)** |
| --- | --- |
| AP2(Real-R) | GGCAGCCAGGTCATAACTCA |
| AP2(Real-F) | CGCACCAACAGATGGGAGT |
| Pds(Real-R) | TTGTACTTGCGGATCTTGG |
| Pds(Real-F) | GCTGGAGCTGGTGTTCG |
| Psy(Real-R) | CATCAAGGATCTGGCGGTA |
| Psy(Real-F) | GGCAGGCATCCACAAGC |
| Ggps(Real-R) | CCCCAGCTCACAGATCACA |
| Ggps(Real-F) | GAGGGAACCCAACAAACCA |
| NEW-SP1 | AGTCCGAGGTGCTGCTCTTGC |
| NEW-SP2 | GCCCTGGAGGAAATGAAGAAAC |
| NEW-SP3 | CTCAGCAACTGGCATGTAGGGA |
| AP2-CHN-BamH I | CGCGGATCCATGCAGGCCTTGCCATTGCATG |
| AP2-CHC-BsrG I | CGCTGTACACTATGATCGCTTTAAGCTTAGG |
| pBI221-N  pBI221-C | TTTAGCCCTGCCTTCATACGC  GACTCTAATCATAAAAACCCAT |
| Actin(Real-R) | TGTCCCTCACAATTTCACG |
| Actin(Real-F) | ATCCTGCGACTAGACCTGG |
