# Supplementary figures and images for: Physiological response of microalga Dunaliella parva when treated with MeJA, GA3
Source: PLoS One. 2024 Oct 22;19(10):e0308730. doi: 10.1371/journal.pone.0308730 (PMC11495637; doi:10.1371/journal.pone.0308730)

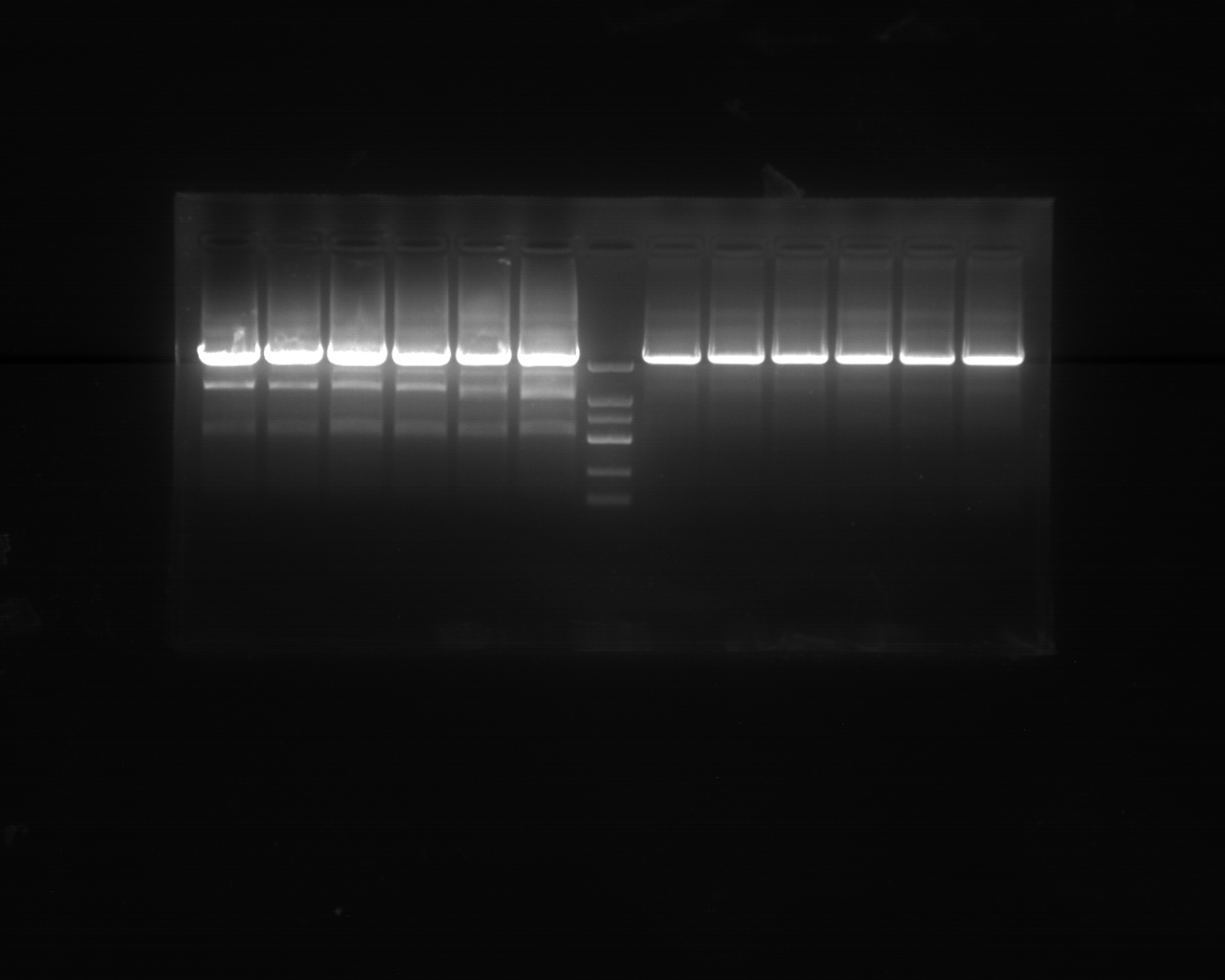

Supplement: S1 Fig — Lane 1 and lane 2 indicate DpAP2 full-length cDNA. Marker indicates DL2000 DNA marker. (TIF) [file pone.0308730.s002.tif]

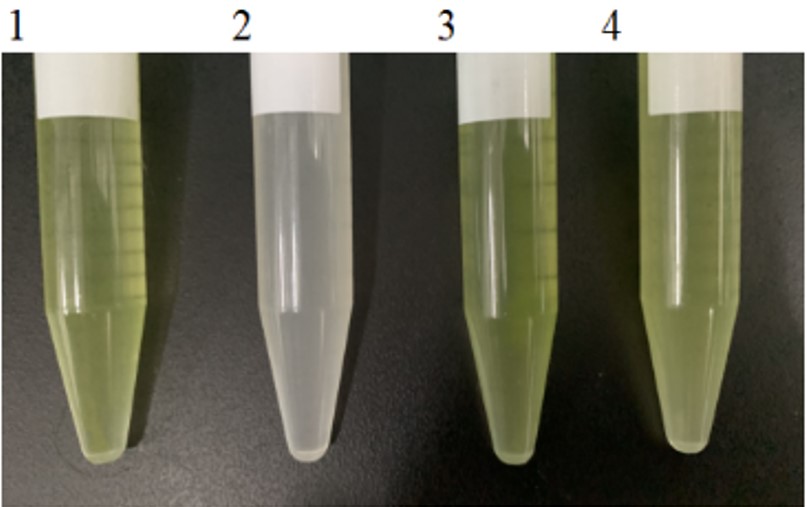

Supplement: S2 Fig — 1: Control group; 2: Control group containing chloramphenicol; 3/4: transgenic D. parva containing chloramphenicol. (TIF) [file pone.0308730.s003.tif]

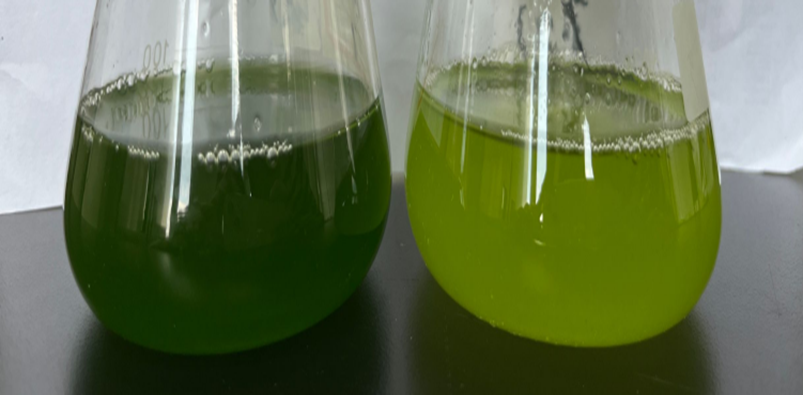

Supplement: S3 Fig — The left culture was transgenic D. parva, and the right culture was control. (TIF) [file pone.0308730.s004.tif]

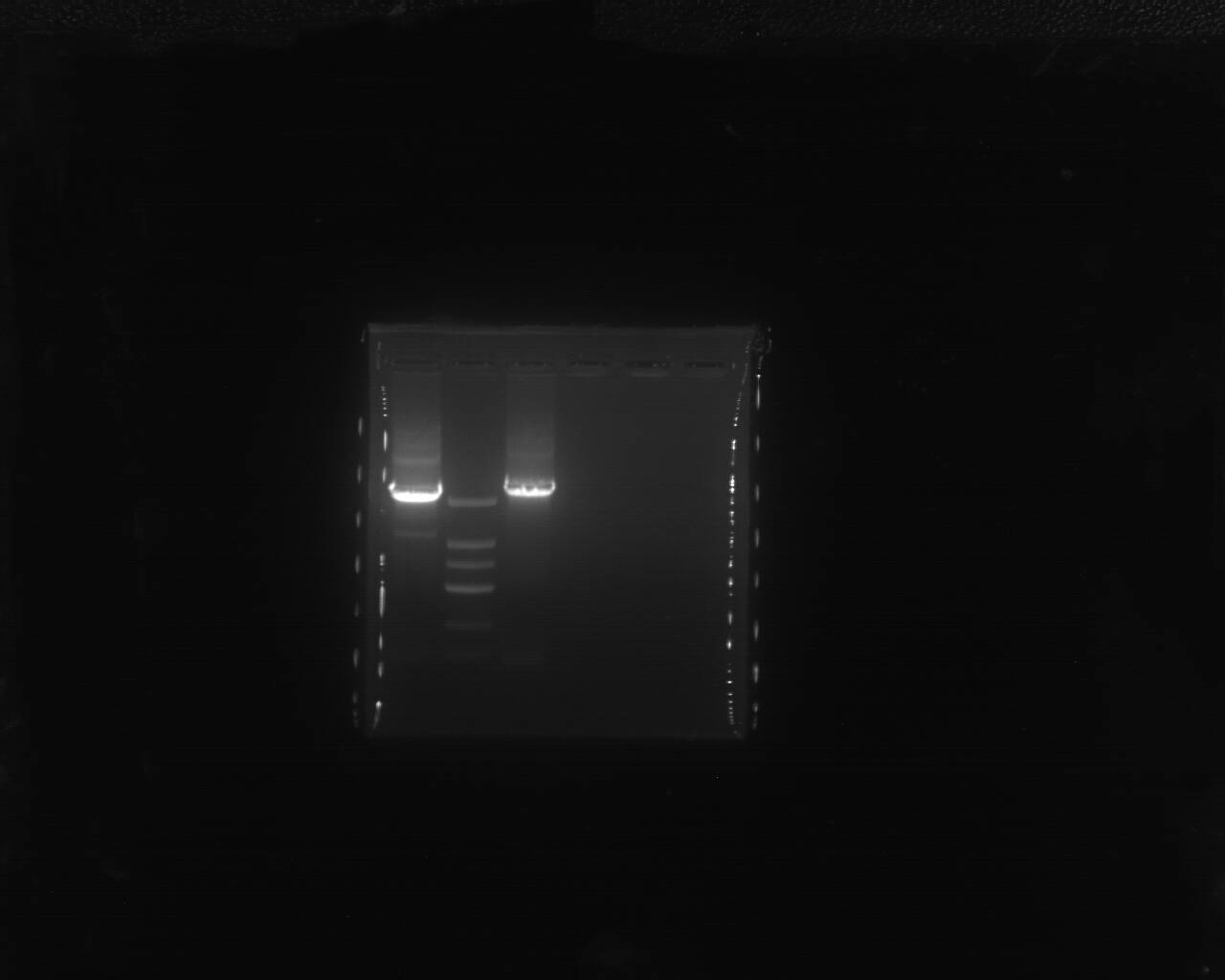

Supplement: S4 Fig — Plasmids were isolated from transgenic D. parva and control as templates of PCR amplification. Lane 1 indicates transgenic D. parva group. Lane 2 indicates control group. Marker indicates DL2000 DNA marker. (TIF) [file pone.0308730.s005.tif]

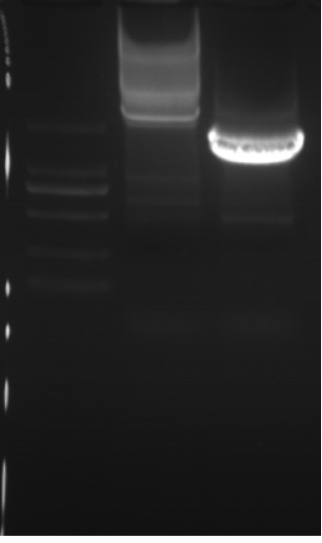

Supplement: S5 Fig — Primers AP1/AP3 are degenerate primers from Genome Walking Kit. Marker indicates DL2000 DNA marker. AP1/AP3 indicate result for second PCR. (TIF) [file pone.0308730.s006.tif]

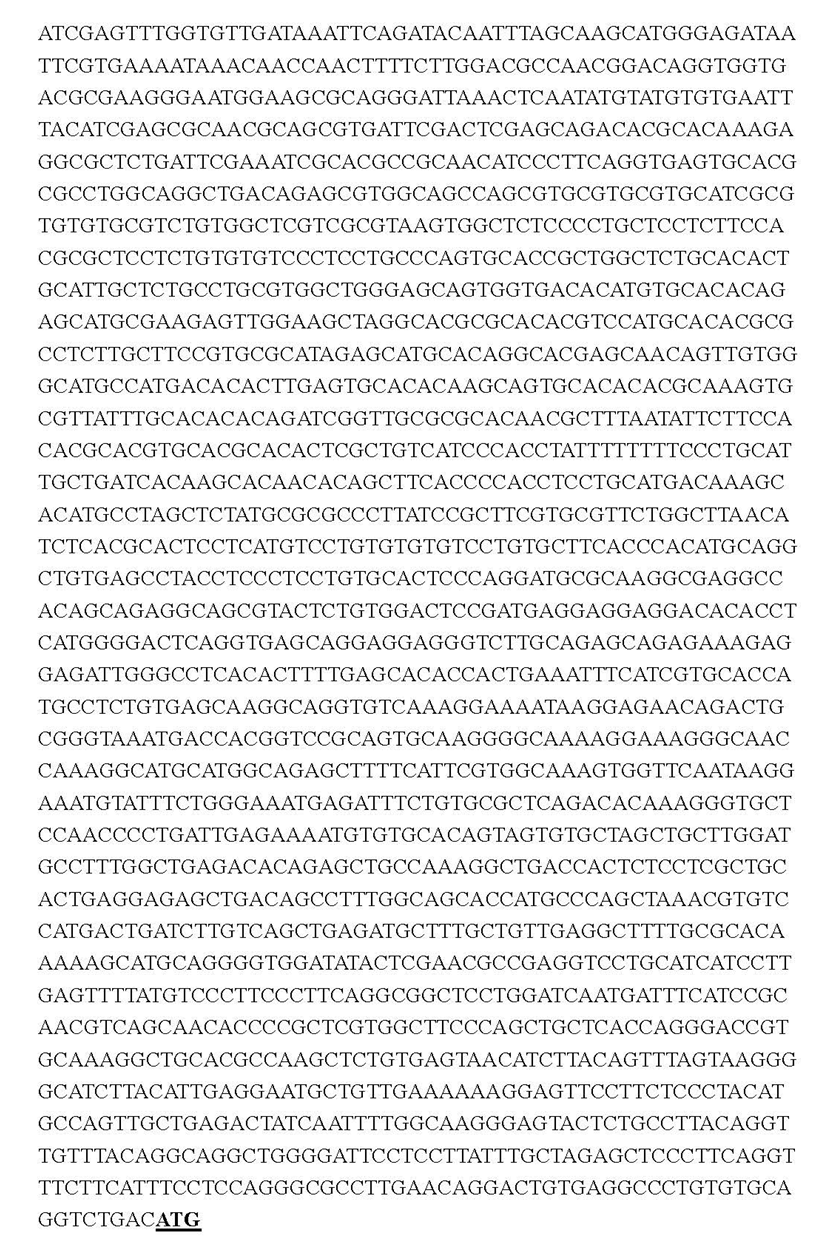

Supplement: S6 Fig — (TIF) [file pone.0308730.s007.tif]
